# Establishment and application of China's first pediatric rational medication intelligent decision system database

**DOI:** 10.3389/fped.2026.1736268

**Published:** 2026-02-04

**Authors:** Dan Wu, Feng Zheng, Yang Chen, Xuhui Zhang, Yin Xu, Hao Chen, Yuxuan Zhou, Wenjie Ni, Xiaobo Zhang, Yi Wang, Zhiping Li

**Affiliations:** 1 Department of Clinical Pharmacy, National Children’s Medical Center, Children’s Hospital of Fudan University, Shanghai, China; 2 Global Health Research Center, Duke Kunshan University, Suzhou, China

**Keywords:** children, database, drug information, medication error, rational medication use

## Abstract

**Objective:**

The incidence of medication errors is much higher among children because of their unique characteristics. This study aims to establish the first Pediatric Rational Medication Intelligent Decision System (PRMIDS) database in China and apply it to the preprescription review system to enhance the safety and effectiveness of medication therapy in children.

**Methods:**

The PRMIDS database was built on authoritative drug information reference books and websites. All drug information was collected, screened, and verified by at least two senior pharmacists. After ensuring consistency with actual local drug use, the information was entered into the system. Based on the severity of potential outcomes, the warning levels of drug information in the PRMIDS were classified into three categories: red (Level 7, absolutely prohibited), yellow (Level 6, relatively prohibited), and green (Levels 1–5, use with caution).

**Results:**

Between November 2018 and June 2025, over 400,000 data entries for 1,030 drugs were integrated. Drug information included conventional dosage, maximum dosage, administration routes, frequency, duplication, drug–drug/drug–food interactions, solvents and compatibility of injections, concentration and administration duration of infusion, allergies, pharmacogenomics, compatibility of Chinese Patent Medicines and Traditional Chinese Medicine, and requirements for decoction. After the PRMIDS database was embedded into the hospital information system, it could automatically obtain the real-time disease status and the latest examination results of children. The implementation of the PRMIDS led to a significant, non-linear reduction in Level 6 prescription alerts. A regression analysis showed a significant decline in Level 6 prescriptions after implementation (*p* < 0.001).

**Conclusion:**

The PRMIDS is the first medication information system specifically designed for Chinese children. This study proves its clinical impact and suggests potential for scalability and generalizability across China.

## 1 Introduction

Children undergo dynamic physiological changes in growth and development. The majority of significant changes in pharmacokinetics and pharmacodynamics occur during infancy and childhood. Growth and development have a significant impact on the process of drug disposition, drug pharmacological effects, and therapeutic outcomes. Therefore, the challenge of rational drug use in children is substantially higher than in adults (1–3). The population of children aged 0–14 in China is approximately 253 million, accounting for 17.95% of the total population (4). How to reduce medication errors (MEs) in children, as well as improve the safety and effectiveness of children's drug treatment, has always been a focus of pediatric medical staff (5–8).

With the development of hospital information systems, Clinical Decision Support Systems (CDSS) (9, 10), especially various types of rational drug use software, have been increasingly developed and applied. Rational drug use software plays an irreplaceable role in handling massive drug information, improving the efficiency of prescription review, controlling the occurrence rate of medication errors and accidents, and reducing the technical risks of doctors and pharmacists (11–14). However, although the mainstream rational drug use software on the Chinese market has relatively complete and practical embedded databases for adult patients, drug information for children remains limited, typically restricted to drug instructions and a small number of guidelines, which do not meet the clinical needs of children's prescription review.

As a national children's medical center, the Children's Hospital of Fudan University has established the first pediatric rational medication information database named the Pediatric Rational Medication Intelligent Decision System (PRMIDS) in response to the lack of pediatric medication databases in China. This system has been developed based on global authoritative databases and tailored to the disease spectrum and medication characteristics of Chinese children. In addition to chemical and biological drugs, it also includes information on Traditional Chinese Medicine and Chinese Patent Medicine. The PRMIDS, independently maintained by our hospital, can conduct personalized and intelligent prescription reviews according to different disease states and biochemical indicators of children within 0.08 s. This computing capability significantly improves the accuracy, efficiency, and homogeneity of prescription and medication order reviews throughout the hospital.

## 2 Materials and methods

### 2.1 Information sources

#### 2.1.1 Printed materials

The main printed reference materials referred to in the construction of the PRMIDS are as follows: drug instructions, Pediatric & Neonatal Dosage Handbook, Chinese National Formulary for Children, Martindale: The Complete Drug Reference, NEOFAX, New Compilation of Pharmacology, and Goodman & Gilman's The Pharmacological Basis of Therapeutics.

#### 2.1.2 Online databases

Online and electronic resources are as follows: Micromedex database (https://www.micromedexsolutions.com), Uptodate database (https://www.uptodate.com), FDA official website (https://www.fda.gov), World Health Organization (WHO) official website (https://www.who.int), LactMed database, and other authoritative information websites.

#### 2.1.3 Drug information retrieval

**Figure 1:**
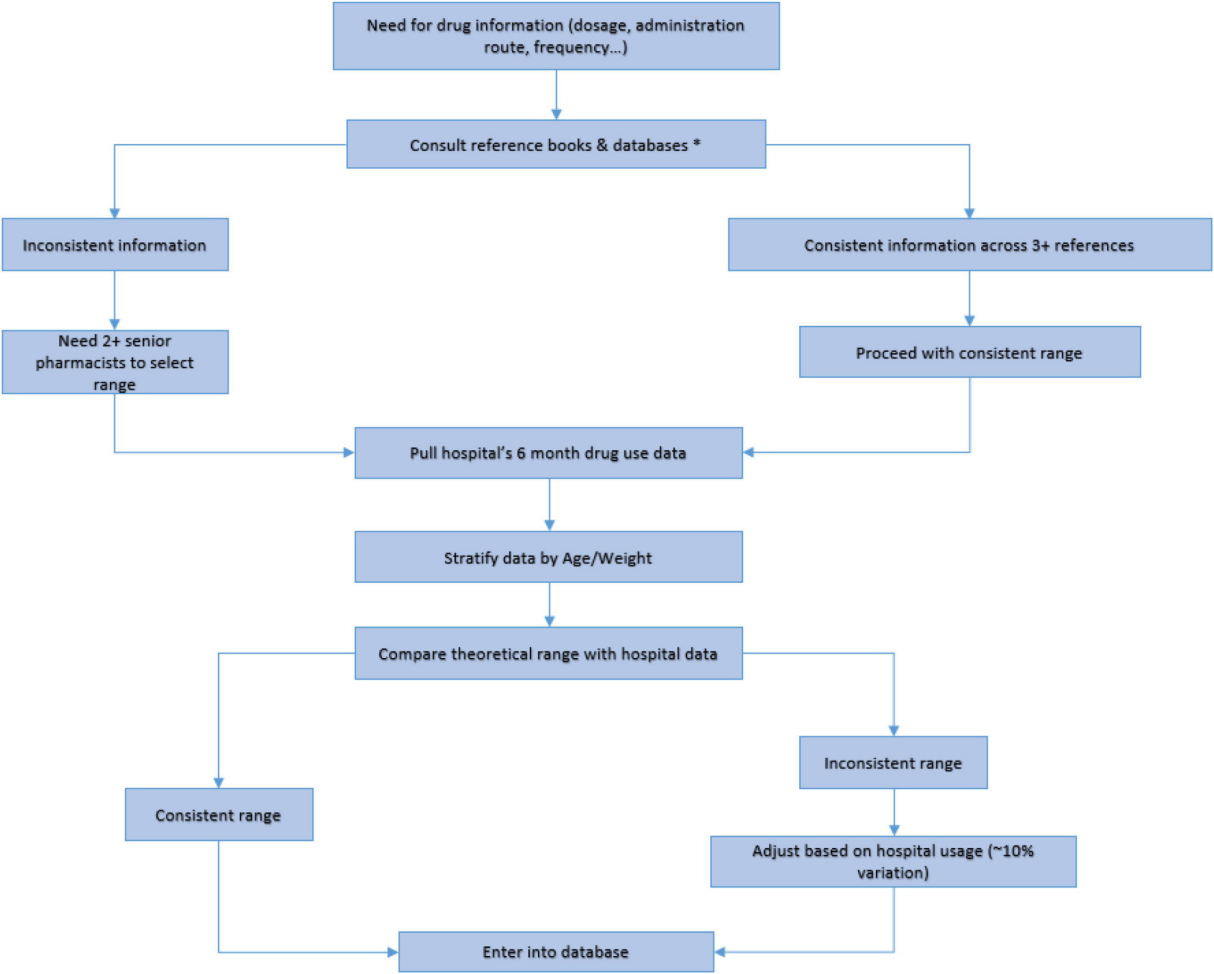
PRMIDS drug information retrieval process *reference textbooks and databases include materials and databases mentioned in sections 2.1.1 and 2.1.2.

The pharmacists involved in the compilation of the knowledge base are senior pharmacists. A senior pharmacist usually holds a PhD degree, has ≥10 years of work experience, and has published at least three academic papers in core journals as first or corresponding author. Senior pharmacists collected and compared drug information from reference books or databases, as explained in Figure 1. All input information was marked with the source of reference material in the system. Before entry, the drug's dosage, frequency, and route of administration history over the preceding 6 months were retrieved from our hospital information system. These real-world data were compared against the reference material to align recommendations with the pharmacologic profile of the Chinese population. Each drug entry required approval by two senior pharmacists. In instances of conflicting information, a predefined evidence hierarchy was followed: first, the Pediatric & Neonatal Dosage Handbook; second, the Chinese National Formulary for Children; and finally, other references. If the discrepancy remained unresolved, a third senior pharmacist was consulted for a final decision.

### 2.2 Warning levels

Based on the severity of potential outcomes, the warning levels of drug information in the PRMIDS are classified into three categories: red (Level 7), yellow (Level 6), and green (Levels 1–5). Common types of drug information for each warning level are given in Table 1.

**Table 1:** PRMIDS alerts classification.

Warning level	Severity level	Clinical scenario	System action
Level 1–5	Mild	Drugs requiring cautious use in pediatric population; hepatic or renal dysfunction; specific administration protocols; or lacking safety and efficacy data in pediatric patients	Automatic pass
Level 6	Severe	Inappropriate^a^ dosage, dosage form, indication, frequency, route of administration, duration of infusion… (above upper limit or below lower limit of regular dose, does not exceed maximums or minimums) Drug interaction labeled as major or severe in the system Drug requires dose adjustment for renal and hepatic dysfunction	Intercepted; pharmacist approval required
Level 7	Life-threatening	Harmful^b^ dose Contraindicated according to patient renal and/or hepatic function Life-threatening allergies or drug interactions Incompatible solvent, prohibited frequency, route of administration Other situations with clear evidence of harm and/or prohibition of use	Automatically blocked, requires physician to change prescription

a Inappropriate: drug dose above or below conventional use limits, but still below maximum dose and above minimum effective dose.

b Harmful: drug dose above maximum limit or below minimum effective dose.

#### 2.2.1 Red drug information (level 7): absolutely prohibited

There is evidence that dosage or administration under Level 7 can cause serious harm to children, and therefore, it is not allowed under any circumstances. For medication orders or prescriptions under Red Drug Information, the system directly prevents submission and requires the doctor to modify such orders or prescriptions.

#### 2.2.2 Yellow drug information (Level 6): relatively prohibited

The dosage under Level 6 exceeds the conventional range and may cause harm to the patient. Therefore, it should be used after weighing the pros and cons. Medication orders under Yellow Drug Information require doctors to fill in the reason for medication, which is then manually reviewed by a pharmacist. If the pharmacist approves the medication order or prescription, it can be submitted normally and can then proceed to the next steps; if not, the pharmacist needs to provide the reason for rejection of the order and must send it back to the doctors, following which the medication order or prescription will be terminated immediately.

#### 2.2.3 Green drug information (Levels 1–5): use with caution

The dosage under Levels 1–5 exceeds the conventional range but has a relatively low risk, and usually does not have a substantial impact on the clinical treatment process of the child. The system only reminds the doctor of the potential medication risks. Medication orders containing Green Drug Information will be directly approved after a prompt window appears at the doctor's end.

**Figure 2:**
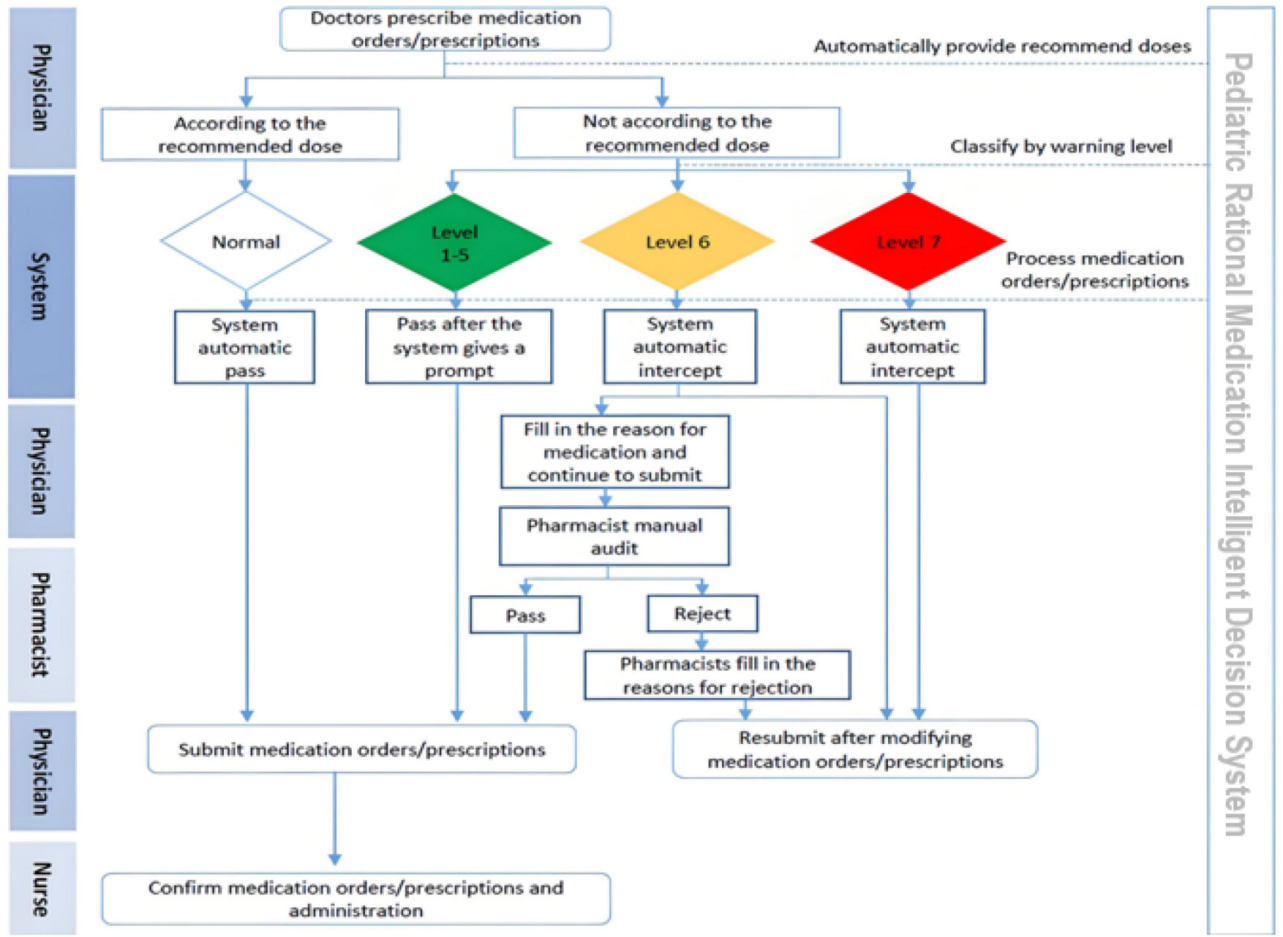
Working process of the pediatric rational medication intelligent decision system.

Overall, the PRMIDS follows a very sophisticated workflow, involving multiple physician–pharmacist interactions in the case of Level 6 alerts. The entire workflow of the PRMIDS is explained in Figure 2.

### 2.3 Internal validation

Internal validation was conducted using a highly configurable, single-simulated patient profile, which was repeatedly adapted to create numerous test cases. This virtual patient was programmed with varying parameters such as age, weight, liver and kidney function levels, and specific disease states, to test medication rules across a comprehensive range of clinical scenarios (e.g., dosing for meningitis vs. peripheral infections). The acceptance criterion for deploying information to the live system was straightforward: the medication rule must execute without any errors or issues when applied to this simulated patient's profile. Successfully meeting this criterion confirmed that the rule was functionally sound and ready for application to real patients within the hospital system.

### 2.4 Statistical analyses

Statistical analyses were performed using IBM SPSS Statistics version 27 (IBM Corp.). Continuous data were presented as mean ± standard deviation or median, as appropriate. Categorical data were presented as frequencies and percentages. Statistical significance was set at *p* < 0.05.

### 2.5 Ethical considerations

The system's development and evaluation were conducted in accordance with the ethical principles in the Declaration of Helsinki. Patient privacy and confidentiality were preserved by using only anonymized and aggregated data for system validation, in full compliance with China's Personal Information Protection Law. While the clinical deployment of the PRMIDS operates under implied consent as part of standard care, any patient data utilized in this research study adhered to the requirement for appropriate informed consent, which was obtained explicitly. To mitigate potential algorithmic bias arising from Western-derived reference ranges, all dosage rules were locally validated against Chinese pediatric prescribing data. The system preserves clinical autonomy by design, functioning as advisory support rather than a mandate, with tiered alerts that require pharmacist consultation for intermediate warnings and that maintain full physician override capability. Finally, we maintained operational transparency, as the underlying logic behind all alerts and their evidence sources is documented and made accessible to clinicians, enabling informed clinical decision-making and allowing traceability.

## 3 Results

### 3.1 Construction of the PRMIDS

**Figure 3:**
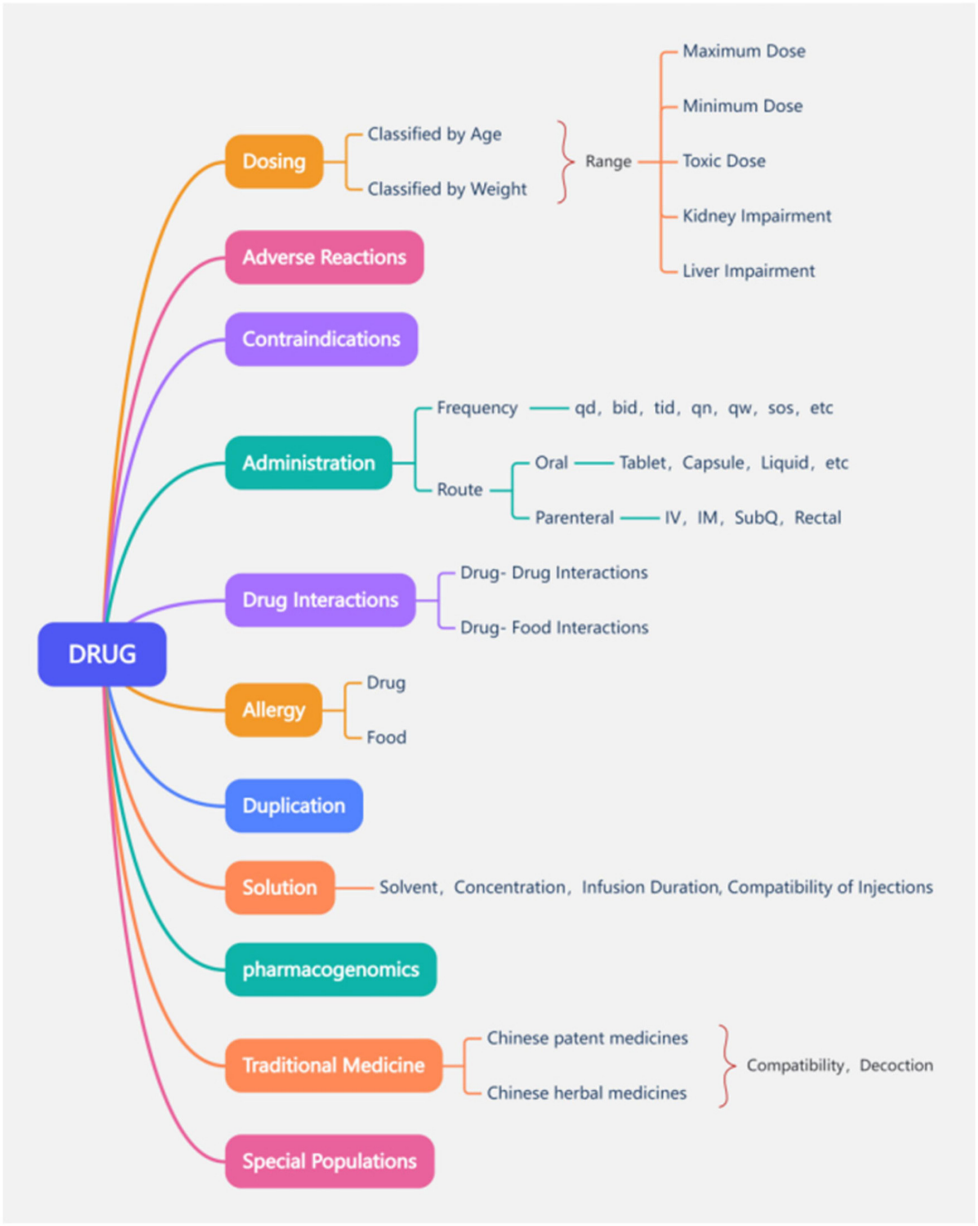
Drug information classification of the PRMIDS database. IV, intravenous; IM, intramuscular; SubQ, subcutaneous.

Since November 2018, over 400,000 data entries for all 1,030 drugs on our hospital's drug list have been maintained. The drug information covered aspects such as conventional dosage, maximum dosage, administration routes, administration frequency, duplication, drug–drug/drug–food interactions, solvents and compatibility of injections, concentration and administration duration of infusion, allergies (drugs and foods), pharmacogenomics, compatibility of Chinese Patent Medicines and Traditional Chinese Medicine, and requirements for decoction, as explained in Figure 3. For highly specialized wards [PICU (pediatric intensive care unit), NICU (neonatal intensive care unit), and oncology] and disease types, more refined and personalized drug information maintenance could be carried out on this basis.

After inputting the drug information in the PRMIDS database, the pharmacist can generate a drug information rule and embed the updated information into the PRMIDS. Pharmacists then perform internal testing through simulation, as explained in section 2.3. After confirming its accuracy, the information is integrated into the hospital system through the PRMIDS and is applied for all patients.

### 3.2 Automatic recommendation of dosage

**Figure 4:**
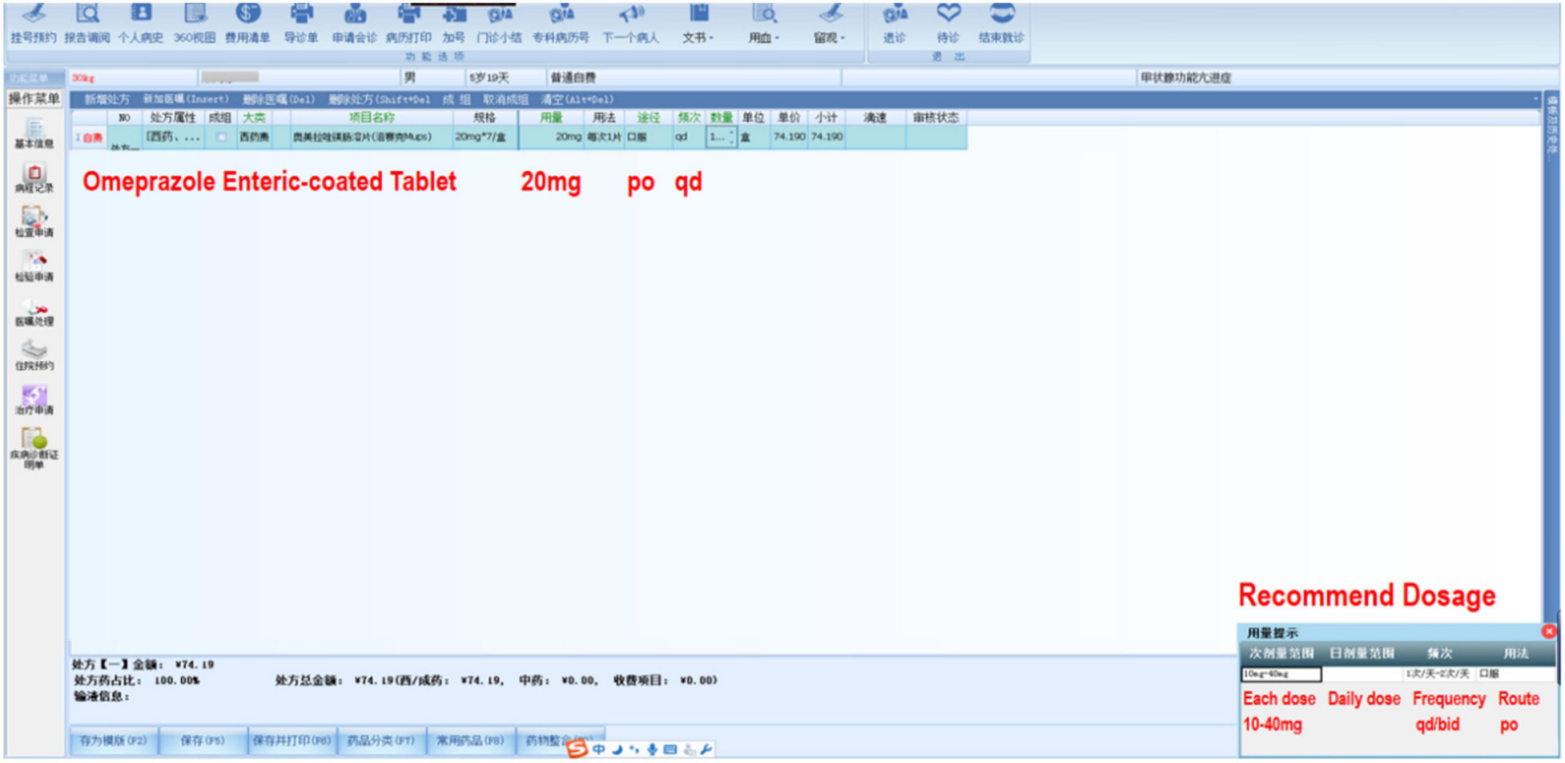
PRMIDS automatic recommendation of dosage.

When doctors write medication orders or prescriptions after entering the drug name in the medication order name column, a drug use prompt box automatically appears in the lower right corner, recommending the conventional dosage, frequency, administration route, single/daily dose range, etc. as a reference for drug use, as shown in Figure 4.

If the dosage, frequency, and administration route entered by the doctor are consistent with the recommended values, the medication order or prescription is successfully submitted to the next step; if the doctor needs to give an unconventional dosage or administration method under certain special conditions, the medication order or prescription may be intercepted according to the rules of warning levels in the system. This provides important help to doctors to correctly prescribe and avoid medication errors caused by memory lapses and calculation errors. If physicians require additional information, they can select the drug's name to view the drug's instructions, guidelines, and other related information.

**Figure 5:**
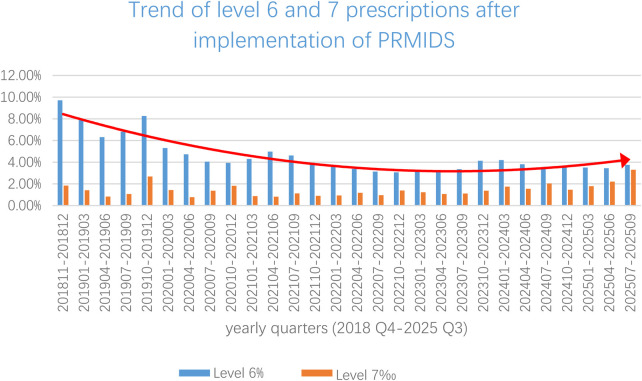
Trends of Levels 6 and 7 prescriptions post-PRMIDS implementation through yearly quarters (2018 Q4–2025 Q3).

The implementation of the PRMIDS led to a significant decrease in Level 6 prescriptions (Figure 5). Temporal trends in Level 6 alert rates were analyzed using both linear and quadratic regression models. The linear model indicated a significant decreasing trend of 0.19% per quarter (*β* = −0.190, *p* < 0.001, *R*^2^ = 0.591). However, a visual inspection suggested a curvilinear pattern, which was confirmed by the superior fit of a quadratic model (*R*^2^ = 0.821 vs. 0.591, Δ*R*^2^ = 0.230). The quadratic model revealed a rapid initial decline of 0.57% per quarter [*β*_linear = −0.569, 95% CI (−0.709, −0.428), *p* < 0.001] that decelerated significantly over time [*β*_quadratic = 0.015, 95% CI (0.010, 0.020), *p* < 0.001]. The inflection point occurred at approximately 19 quarters after implementation (mid-2023), after which the rates stabilized around 3.7%, representing an optimal performance baseline.

**Figure 6:**
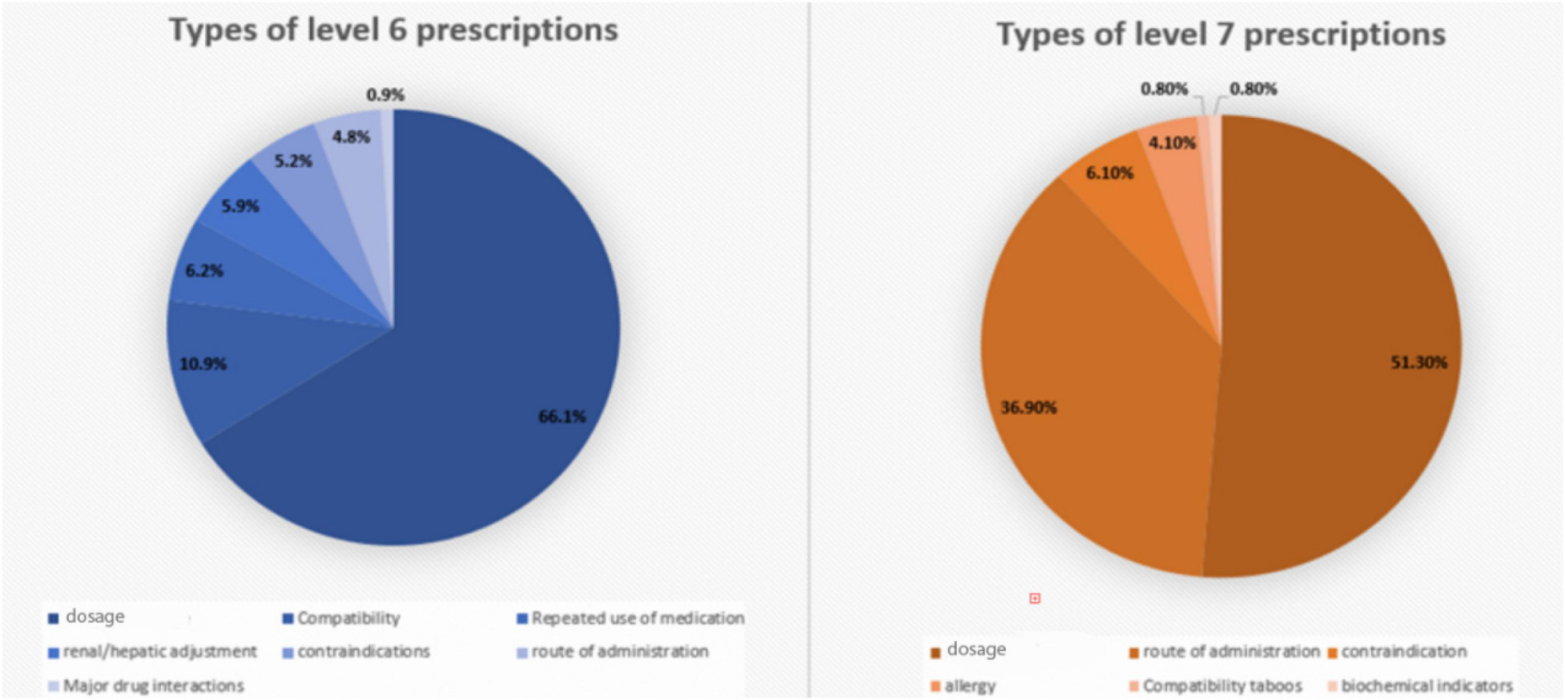
Types of Levels 6 and 7 prescriptions (2018–2025).

Level 6 and 7 prescriptions intercepted by the PRMIDS also varied, with dosage errors representing the majority of errors, 66,1% and 51,3% for Levels 6 and 7 alerts, respectively. Figure 6 shows the types of Levels 6 and 7 prescriptions collected between 2018 and 2025.

### 3.3 Automatic dose adjustment based on biochemical indicators

**Figure 7:**
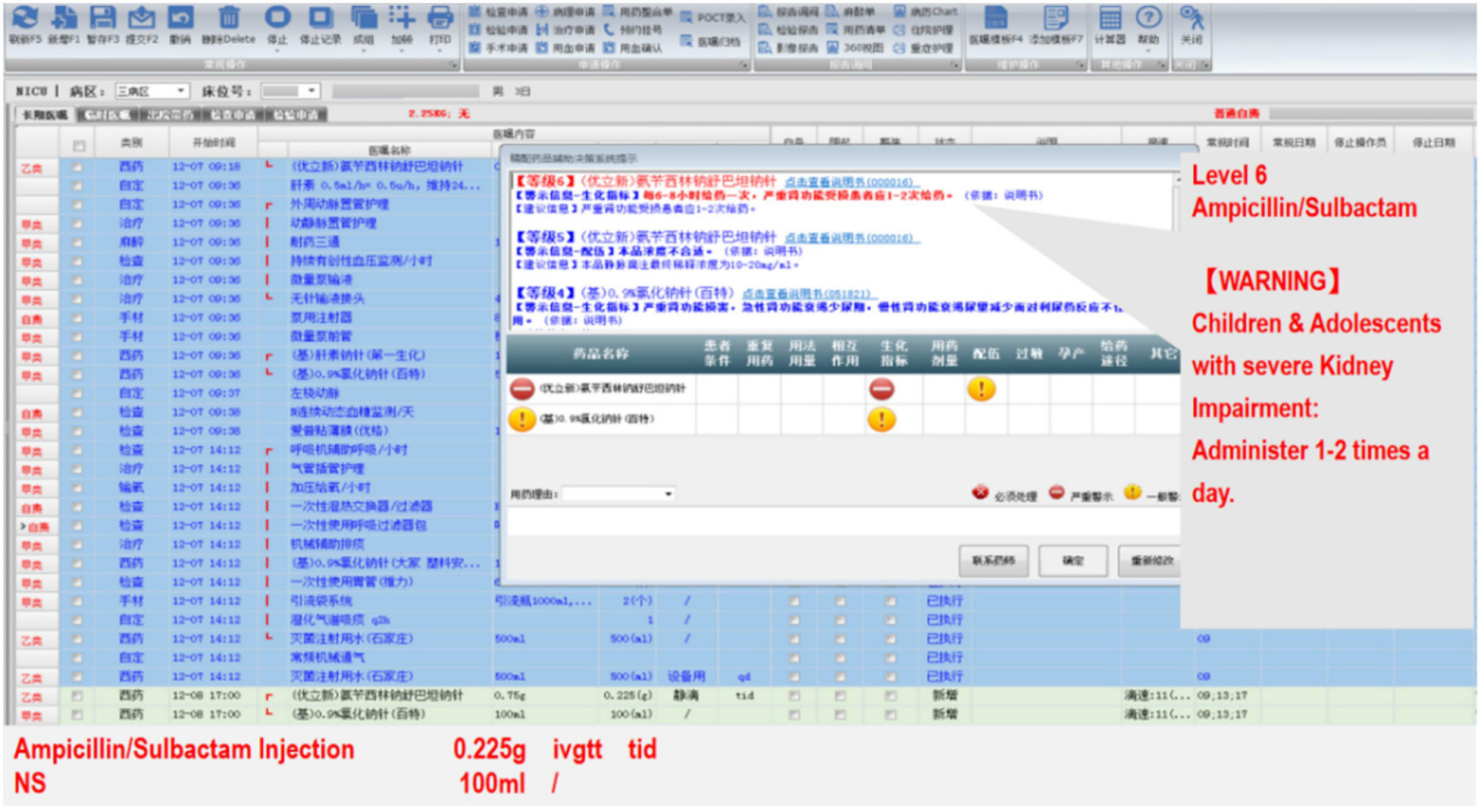
PRMIDS automatic dosage adjustment based on biochemical indicators.

The information in this database is also stratified according to the different disease states and biochemical indicators of children. After the PRMIDS is embedded into the hospital information system, the PRMIDS automatically retrieves the real-time disease status (such as clinical diagnosis and body temperature) and the latest test and examination indicators (such as hepatic and renal function and blood routine and bacterial culture test results) of pediatric patients through other hospital information platforms, as shown in Figure 7. Based on the pre-entered information (such as meningitis/non-meningitis status and endogenous creatinine clearance rate value), the system automatically provides recommended target dosage to doctors and intercepts unreasonable prescriptions at the same time.

For example, for a 5-year-old child, the system calculated the patient's creatinine clearance rate as 25 mL/min/1.73 m^2^ based on the latest renal function. When the doctor prescribed a conventional dosage of cetirizine drops of 0.25 mL, bid, as an antiallergy drug, the system intercepted the entry and indicated that the child had severe renal function impairment. It then recommended that the dose be lowered to 0.125 mL, qd for treatment. Another example: a child was diagnosed with bacterial meningitis, the doctor prescribed meropenem at a standard sepsis dose of 20 mg/kg q8 h for anti-infective treatment, and the system intercepted the entry and suggested 40 mg/kg, q8 h for bacterial meningitis.

### 3.4 System performance

The PRMIDS was designed to provide real-time, point-of-care decision support. System responsiveness was benchmarked during development, with engineering tests indicating an average rule evaluation time of approximately 0.08 s (range: 0.06–0.10 s) under simulated clinical load. This ensured that the alert process was imperceptible during order entry.

### 3.5 Real-time communication

Through the PRMIDS's built-in instant messaging system, doctors can get in touch with online prescription pharmacists at any time (ward pharmacists during business hours 08:00–17:30, on-call pharmacist 17:30–08:00). Both doctors and pharmacists can contact each other through the system, with the option to attach pictures, links, and files to their text messages.

In addition, doctors can contact pharmacists by entering the reason for a particular medication in the prescription review interface or by calling the clinical pharmacy department, which is available 24/7. For some clinical drug treatment plans that are not mentioned in the existing drug information database, if the doctors provide relevant evidence-based materials to support their claims, pharmacists will evaluate and decide whether to incorporate the treatment plans into the information base.

### 3.6 Feedback and continuous improvement

In addition to updating physician-initiated PRMIDS database information, pharmacists regularly review doctors’ orders and prescriptions, communicate with the clinical department about general and typical problems in the prescription review process, and check the latest drug information and guidelines to update the content of the knowledge base in a timely manner. For problems that cannot be solved through simple communication, pharmacists record and collect them in a special “drug-related problems” document, which is then brought up and discussed in multidisciplinary meetings. The pharmacy department also organizes regular discussions with experts from the hospital's medical department, ethics committee, and related clinical departments, to continuously improve and refine the knowledge database of the PRMIDS according to the discussion results, so as to make it more in line with the actual clinical needs.

## 4 Discussion

### 4.1 A need for a better system

Because of their inherent particularity and complexity, children are more prone to MEs during the initiation of drug therapy. The main causes of these errors are a lack of standard pediatric dosages (15, 16), calculation mistakes during dosage conversion (17), the use of non-pediatric formulations, and inappropriate administration routes (18, 19). According to the statistics of the WHO, the incidence of medication errors in children is three times that of adults (7, 20). It is reported that 10%–72.7% of patients in pediatric emergency departments (PEDs) have experienced medication errors, with severe errors accounting for 18.1% of all MEs. About one-third of patients have experienced at least one medication error (21, 22). Reducing medication errors, ensuring medication safety, and improving the quality of pharmaceutical care remain key objectives of global pediatric medical professionals.

CDSS are widely used in various fields such as medical services, public health emergency management, hospital management, and medical insurance management because of their ability to leverage computer and network communication technologies, especially new technologies such as data warehousing, data mining, online analytical processing, and artificial intelligence. These technologies enable rapid identification, capture, and integration of information to form an optimized combination scheme for decision-makers (10–13, 23). Among them, the development and application of rational drug use software is becoming increasingly widespread. Rational drug use software plays an irreplaceable role in handling massive drug information, improving the efficiency of prescription review, controlling the incidence of medication errors and accidents, and reducing the technical risks of doctors and pharmacists.

Computerized Physician Order Entry (CPOE) (24–26), Drug Therapy Screening System (DTSS), and Australia's Electronic Prescribing System (Eprescribe) (27, 28) are commonly used rational drug systems. However, because of differences in disease spectra, therapeutic regimens, language barriers, and system compatibility, these foreign systems have not been widely adopted in China. Domestically, mainstream systems such as the Prescription Automatic Screening System (PASS) (29) and Drug Consultation & Safety Monitoring System are more prevalent in general hospitals owing to their powerful embedded databases and user-friendly interfaces.

However, although rational drug use software has relatively complete and practical embedded databases for adult patients, it is lacking in pediatric medication information. It usually includes only drug instructions and a limited number of guidelines, which are inadequate to meet the actual medication needs of pediatric patients across different age groups, disease states, and pathophysiological conditions. Therefore, as a national children's medical center, our hospital, taking advantage of the opportunity to build a hospital information platform, organized senior clinical pharmacists and prescribing pharmacists to establish the PRMIDS.

### 4.2 PRMIDS in the context of pediatric CDSS: comparison with existing systems

The PRMIDS aims to fill gaps that other software cannot and offers a user-friendly interface focused on the Chinese population and their specific needs in contrast to other widely used support systems. As opposed to other CDSS, which offer a more generalist and one-size-fits-all solutions, the PRMIDS is built not only for the pediatric population, but also for the Chinese pediatric population, which allows it to offer highly individualized and personalized care, especially with the integration of the patient's clinical data as part of the system's workflow. In addition, while other systems are usually built in the English language and offer Chinese user interface, the PRMIDS is made fully in the Chinese language, which reduces physician strain and is naturally more user-friendly in the Chinese context. Another important aspect of the PRMIDS is its inclusion of Chinese Patent Medicine and Traditional Chinese Medicine, which allows a complete drug information review as opposed to other Western systems, since Traditional Chinese Medicine is widely used in our healthcare system.

Therefore, we believe that the PRMIDS could meet national pediatric medication needs in response to the lack of pediatric rational medication databases in China.

### 4.3 System features and technical implementation

The operating system of the PRMIDS is developed by Xiamen Jingpei Software Engineering Co., Ltd., Fujian, China. The operation interface is divided into five core modules, namely outpatient/inpatient medication order real-time review, prescription and medication order query, outpatient/inpatient prescription evaluation, medication rule maintenance, and system settings. The input and maintenance of drug information was completed under the medication rule maintenance module. Another core module, outpatient/inpatient medication order real-time review, was customized according to the characteristics of patients in our hospital. We removed adult-specific clinical fields such as dental and craniofacial implants and obstetric and gynecological history from the visual interface and added clinical fields relevant to pediatric patients, such as gender, age, height, weight, body surface area, allergy, TBIL, DBIL, ALT, AST, CRES, UREA, ALB, WBC, NEUT%, and CRP to allow pharmacists to quickly obtain information about pediatric patients. If the aforementioned information is still insufficient to assist pharmacists in evaluating a prescription, they can select the child's name to enter the integrated interface of the hospital information system, and through this interface, access subinterfaces to assist pharmacists in comprehensively understanding the patient's condition.

**Figure 8:**
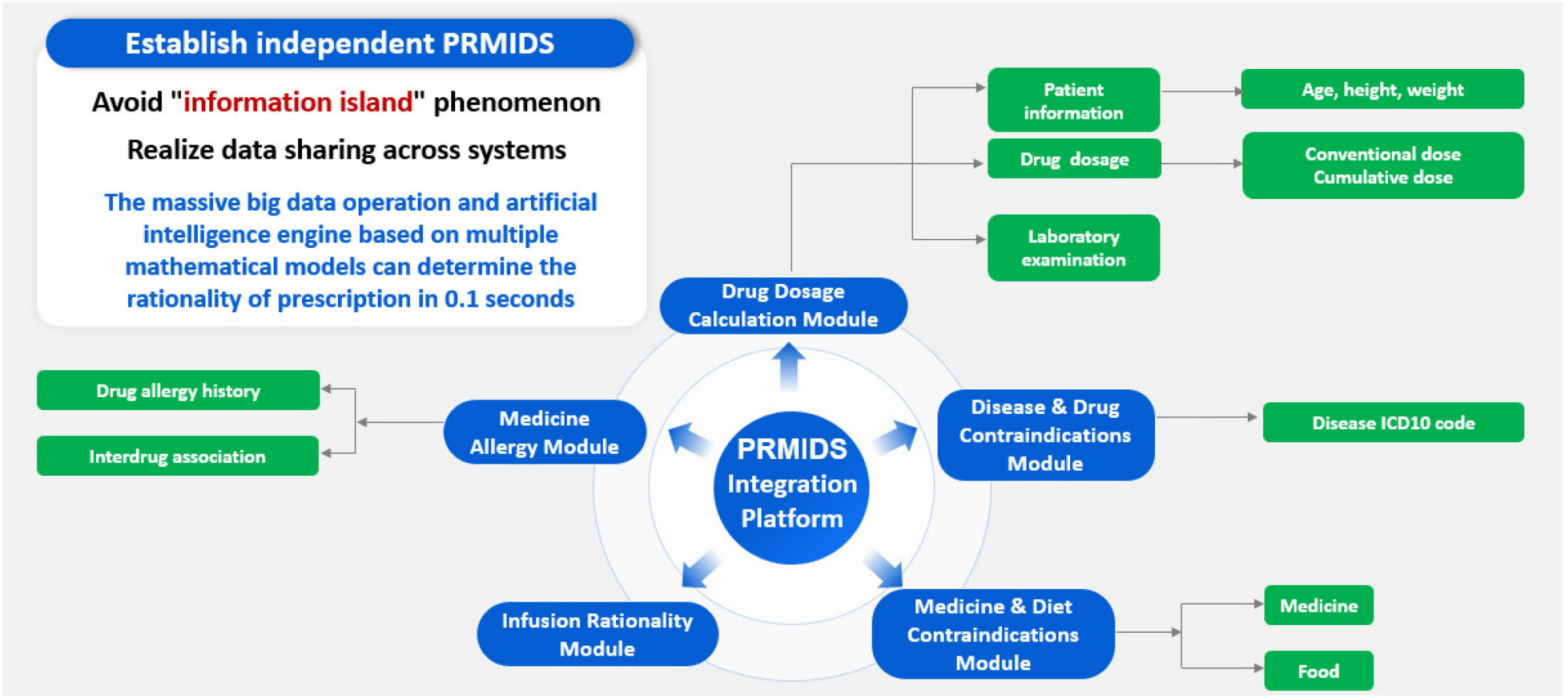
Operation mode of the PRMIDS integration platform.

Through database maintenance and interface optimization, the PRMIDS can innovatively provide automatic and individualized recommendations for the patient and intercept medication error based on the patient's actual physiological and pathological conditions. The foundation for realizing these two functions is that the PRMIDS can obtain accurate patient information from the hospital information management system, including the patient's basic information, laboratory test and image results, disease-related information, and medication order issuance and discontinuation information. Such information needs to be correctly uploaded to the hospital information management system by different subsystems before it can be called up. At the initial stages of the establishment of the PRMIDS, taking advantage of the opportunity of the construction of Healthcare Information and Management Systems Society (HIMSS) Stage 7 (30, 31), all subinformation platforms in the hospital achieved information interconnection and interoperation, so that pharmacists could acquire and access information from other subplatforms unrestrictedly. Ultimately, within 0.08 s, as benchmarked by the system's engineers, the PRMIDS subsystem could make personalized recommendations and finish a patient's prescription review. Figure 8 explains the overall technical architecture and operational workflow of the PRMIDS.

### 4.4 Observed impact and workflow integration

By stratifying the safety of orders and prescriptions, pharmacists only need to manually review Level 6 orders, which are relatively complex and require a risk–benefit assessment. For easier-to-judge orders (Levels 1–5 and 7), the system can make judgments and choose to intercept or release them automatically. It is important to emphasize that the PRMIDS is a system designed as decision support and not as a replacement. Its main goal is to reduce human errors by integrating a rule-based system. However, physicians still maintain the right to override the system's decision, with the approval of a pharmacist.

The prereview of prescriptions and orders has greatly reduced the pressure on traditional manual prescription review for pharmacists. Since the integration of the PRMIDS in our hospital, the number of Level 6 orders and prescriptions needing manual review decreased to approximately 4% of the total number of prescriptions, averaging over 7,000 per month, as shown previously in Figure 5. Pharmacists can now focus more on identifying and reviewing problematic orders, thus improving work efficiency.

By automatically providing doctors with recommended doses and assisting pharmacists in prescription prereview, the PRMIDS greatly reduces the requirements of medical staff on medication knowledge reserve, improves the level of medical homogenization, and releases the medical staff's workload. It also mitigates the potential risk of medication errors caused by dose conversion and enhances the level of clinical medication.

### 4.5 Implementation challenges

As with any major change in hospital management and workflow, the integration of the PRMIDS in our hospital was met with a few but expected challenges. First, the integration period, which lasted around a year, required extensive customization and testing to interface with our existing hospital information systems and ensure reliable, real-time data exchange. Second, the cost of implementation was roughly $70,000, which covered software licensing, interface development, and initial training. Third, as with other existing systems, alert fatigue was one of our main concerns, which we attempted to mitigate with alert stratification (Levels 1–7), and by ensuring that only critical (Levels 6 and 7) alerts would disrupt workflow. Last, establishing a dedicated, multidisciplinary maintenance team, including hospital and clinical pharmacists, IT specialists, and pediatricians, was crucial for ongoing system updates, troubleshooting, and ensuring the long-term clinical accuracy and relevance of the decision rules.

### 4.6 Implementation and generalizability

The successful implementation of the PRMIDS in our tertiary, HIMSS 7-certified center provided a robust proof of concept, demonstrating strong clinical and economic benefits, including the reduction of medication errors. To translate this success to broader settings, a strategic, phased approach is essential. Therefore, we have planned a three-stage expansion, from internal branch hospitals to a national network, to manage scalability. For community hospitals with fewer resources, deployment can be adapted through modular implementation (starting with high-risk drug modules), cloud-based hosting to minimize local IT burdens, and tiered subscription models to align with institutional size and budget. Key implementation considerations include upfront integration, ongoing maintenance requiring clinical pharmacists for rule updates, and targeted training for pharmacists and physicians. The ultimate goal is to extend the PRMIDS to over 50 hospitals, serving more than 10 million children over the next 3 years.

### 4.7 Limitations

This study and the current iteration of the PRMIDS have several important limitations. First, the evaluation framework is incomplete. As a study focused on system establishment and initial clinical outcomes, we did not conduct structured user acceptance testing or collect systematic clinician feedback on usability and perceived utility. Consequently, we lacked quantitative data on critical human–computer interaction metrics, such as false-positive alert rates, physician override patterns, and user satisfaction, which are essential for optimizing system specificity and minimizing alert fatigue. Second, the system's knowledge base and technical design have inherent constraints. While derived from authoritative sources, the system is updated on a quarterly basis, creating a potential lag between new evidence publication and clinical implementation. Furthermore, the rule-based, deterministic architecture lacks the adaptive learning capabilities of machine learning models and may have limited coverage for specialized populations such as oncology patients, those with rare diseases, or transplant patients, although this limitation is currently being mediated as more rules are being integrated into the system. Third, system performance is contingent on external data quality. The accuracy of personalized recommendations (dose adjustments based on renal and hepatic function) depends entirely on the correctness and timeliness of data entered into upstream hospital systems (laboratory results and diagnoses). Finally, the context of development affects generalizability. The system was implemented and validated within a resource-rich, tertiary national children's medical center with advanced HIMSS 7-level data integration. Its effectiveness and the required implementation resources may differ in community settings with limited resources. These identified gaps, particularly in user-centered evaluation, alert precision analytics, and coverage of complex subpopulations, define clear priorities for future research and system iteration.

## 5 Conclusion

The construction and continuous optimization of the PRMIDS has been the result of the wisdom and diligence of the pharmacists at our hospital. Since its clinical application, the PRMIDS has played a positive role in many ways, such as handling massive drug information, improving the efficiency of prescription review, controlling the occurrence rate of medication errors, reducing the technical risks for doctors and pharmacists, enhancing the homogeneity of medical care, and promoting safe and rational medication use. Moreover, because of the absence of language and system compatibility issues, the software was more suitable for promotion and application in children's specialized hospitals as well as maternal and child healthcare institutions across various regions in China, benefiting more Chinese children.

## Data Availability

The original contributions presented in the study are included in the article/Supplementary Material, and further inquiries can be directed to the corresponding authors.
